# Satisfaction With the Self-Assessment of University Students Through *e-Coping With Academic Stress Utility*^TM^

**DOI:** 10.3389/fpsyg.2018.01932

**Published:** 2018-11-08

**Authors:** Jesús de la Fuente, José Manuel Martínez-Vicente, Francisco Javier Peralta-Sánchez, María Carmen González-Torres, Raquel Artuch, Angélica Garzón-Umerenkova

**Affiliations:** ^1^Department of Theory and Methods in Education and Psychology, School of Education and Psychology, University of Navarra, Pamplona, Spain; ^2^Department of Psychology, Universidad Autónoma de Chile, Santiago, Chile; ^3^Department of Psychology, University of Almería, Almería, Spain; ^4^Universidad Internacional de la Rioja, Logroño, Spain; ^5^Fundación Universitaria Konrad Lorenz, Bogotá, Colombia

**Keywords:** SLPS Competency Model, university academic stress, e-technological development, innovation transfer, validation study

## Abstract

The general purpose of this report is: (1) research was to check whether the degree of satisfaction with the self-assessment activity of university students was related to the scores obtained and the degree of different variables, associated with level of Self-Regulation; (2) to present the online utility, *e-Coping with Academic Stress*^TM^, as a technological development in Educational Psychology; (3) analyze the possibilities of transfer of this technological innovation. A total of 929 university students, coming from a public university, participated in the use of this online utility. University students can use the tool’s online inventories to make self-assessments in the different variables of Studying, Learning and Performing under Stress (SLPS Competency Model). Descriptives, correlational and inferential analyzes (ANOVAs and MANOVAs) were carried out. The results allowed to know the profile of competences of the analyzed university students, in addition to the degree of satisfaction with the self-evaluation. Finally, we communicate possible actions and options available for transfer of this resulting technology, through RD transfer contracts arranged directly or with other universities.

## Introduction

The value chain of RD & I (Research, Technological Developent, and Transfer of Innovation) in Educational Psychology is conceptualized as carrying out scientific research, for the production of technological development (processes, products or services) that ultimately gives rise to innovation (and its transfer) as the ultimate element of the process. The RD & I value chain can mean an advantage to the different activities of academics, research, and professional practice, with respect to processes, products and services that are generated in the sphere of psychology and education (de la Fuente et al., 2018a). Consistent with the previous conception, the purpose of this report is: (1) research was to check whether the degree of satisfaction with the self-assessment activity of university students was related to the scores obtained and the degree of different variables, associated with level of self-regulation: (2) to present the online utility, *e-Coping with Academic Stress*^TM^, as a technological development in Educational Psychology; (3) analyze the possibilities of transfer of this technological innovation.

The previous questions that this research has guided are: on what variables does the degree of satisfaction depend on the self-assessment of the students?, Does it depend on the degree of personal self-regulation?, Are there some variables that determine the degree of satisfaction positively or negatively?

### The Problem of Stress as Academic Emotion

Human response to stress has been studied extensively in several contexts, especially in clinical and healthcare fields (O’Donovan and Hughes, 2007; Hamdan-Mansour et al., 2009; Pettit and De Barr, 2011; Costarelli and Patsai, 2012; Gulewitsch et al., 2013; Schönfeld et al., 2017). In the educational field, however, despite significant progress in understanding cognitive and metacognitive processes, more effort is required to gain a clear understanding of the mechanisms that make up the stress response, especially as the negatively impacts these processes (Pintrich, 2004; de la Fuente, 2014a). Stressful study situations are learning and performance contexts that can trigger stress responses (cognitive, physiological and motor). This is particular true when pursuing a university degree or competing for employment through professional exams. If stress responses are severe, learning or performance may suffer. Such high-stress contexts are likely to appear over the course of academic and professional life for virtually every university student or graduate.

Recent research has revealed the importance of taking into account positive and negative emotions experienced during learning and performance processes at university (Andersson et al., 2009; Bardi et al., 2011; Hamaideh, 2011; Postareff et al., 2016; Pekrun et al., 2017). Metaphors of information processing and construction formed the basis of classic, first-generation cognitive models, and not enough attention was given to emotions and how they affect cognitive processes while learning. After this, motivational-affective models appeared as the second generation (Pintrich, 2004; Zimmerman, 2008), and insisted on the need to pay attention to and intervene in affective processes that operate during learning, because they affect and accompany the cognitive processes that support information processing, sometimes positively and sometimes in a negative, interfering way. Negative emotional experiences can take place when learning under stress at the university, and even more so when preparing for professional exams; this process must become fully understood (Regehr et al., 2013).

Evidence shows that, during the process of learning and study, the different manifestations of a stress response interfere in cognitive and motivational processes (Serlachius et al., 2007; Chou et al., 2011). We must understand the stress response if we seek to understand learning processes in general, and particularly study processes. Stress has been shown to interfere with processes of memory, attention, and information recall. The anxiety associated with stress can lead to more and more worry and negative emotionality. When negative thoughts and irrational beliefs replace positive thinking, a weakened motivational-affective state ensues, and demotivation follows (Largo-Wight et al., 2005).

Consequently, effort must be made toward prevention of stress responses, students must be helped toward establishing the competencies of managing stressing and being ready for its appearance. However, this aspect is usually overlooked in preparatory programs for university students and exam candidates (Conley et al., 2013), which focus almost exclusively on academic content.

### Theoretical Foundation

#### Model of Competency for Studying, Learning and Performing in Stressful Contexts

The *Competence for Study, Learning and Performance with Stress* model (SLPS Competency) is a multi-dimensional construct (de la Fuente, 2015a) inasmuch as it refers to stress factors for university students and exam candidates, and to stress management. This model is based on the 3P model (presage-process-product) of Biggs (Biggs, 2001) and orders the variables to be analyzed within it. The *presage* variables refer to the predictor variables or previous experience of the student, which is not modifiable. The *process* variables are mediating variables and refer to the level of competence to studying, learning and performin inder stress situations (CSLPS model). The *product* variables refer to the level of stress experienced in the learning situation.

##### Presage variables: past experience

This variable refers to how a subject assesses his or her past experience with professional examinations, inquiring into the subject’s perception of examinations past, along with present and future expectations. Looking at university populations, recent reviews have analyzed factors that mediate between academic self-efficacy, created from past experiences, and later academic performance (Honicke and Broadbent, 2016), as well as the effect of prior experiences on creating expectations (Jones, 2017).

##### Process variables: competence for studying and learning under stress

This type of competence evidently does not refer to a single skill or to one type of knowledge. The individual constructs a set of skills, knowledge, attitudes and habits that enable him or her to confront a certain assessment situation and succeed. The *Multidimensional nature of the Competency for Studying, Llearning and Performing under Sstress, CSLUS* (de la Fuente et al., 2013b; de la Fuente, 2015a) is characterized by assuming three levels of learning:

(1) Knowledge:
*Facts* (knowledge about the characteristics of the class subject or professional exam: job openings, percentage of candidates who pass, requirements)
*Concepts* (competitive exam system, requirements; type of examination, scoring, prior merits/credits, type of class subject)
*Principles* (beliefs about the professional exam or selection process)
(2) Know how:
*Instrumental skills* (written and oral skills; control over anxiety)
*Learning and study skills* (study skills and techniques)
*Cognitive meta-skills* for study (learning strategies), *emotional meta- skills* (coping strategies) and *behavioral meta-skills* for managing stress (self-regulation strategies)
(3) Mindset:
*Attitudes* and values (academic behavioral confidence, achievement motivation)
*Study habits* (time management, persistence, discipline)

##### Product variables: positive vs. negative emotions

*Engagement* has been defined as the emotional involvement that accompanies an intense experience. On one hand, it can be promoted by the dynamics of a situation or the immediate context, which creates situational interest (Hidi and Anderson, 1992). On the other hand, it refers to a multidimensional construct that involves three dimensions: (1) cognitive engagement (self-regulation); (2) behavioral engagement (putting forth effort); (3) emotional engagement (interest) (Friedricks et al., 2004).

Maslach et al. (1996) conceptualized the *burnout* síndrome as a response to chronic work stress, including a feeling of emotional exhaustion, attitudes of depersonalization (negative feelings toward the people that one works with), and a lack of personal fulfillment in work, through the appearance of processes that devalue one’s own professional role. Recent research has offered evidence that the burnout syndrome in students comprises three dimensions (Shaufeli et al., 2002): (1) Exhaustion: A feeling of fatigue caused by academic demands; (2) Cynicism: A cynical, distant attitude regarding academic tasks; and (3) Perceived competence: Feeling ineffective as a student. In university students, burnout is manifest primarily through emotional fatigue, while manifestations of cynicism and lack of personal fulfillment are scarce in these populations. The importance of level of emotional fatigue in university students stems from its role as a modulating variable that significantly influences students’expectations of successfully completing their studies.

#### Academic Self-Assessment From the Theory of SRL vs. ERL

*Self-assessment* is a behavioral activity that forms part of self-regulating behavior (Brown, 1998) and self-regulated learning (Zimmerman and Labuhn, 2012). In the academic sphere, it is an essential element of self-knowledge and self-improvement. Different repertories are involved: knowledge (knowing what to assess), skills (knowing how to assess) and attitudes (wanting to self-assess). From this point of view, the e-coping utility is a technological development that places these elements at the university student’s disposal: (1) students are offered knowledge of the variables for self-assessment and better self-understanding, in order to study and learn under stressful conditions; (2) they are given a procedure so they know how to self-assess each variable, as well as information about levels of competence in each; and (3) a satisfactory experience with self-assessment is made possible (de la Fuente et al., 2016). According to *Self-Regulated vs. Externally Regulated Learning Theory*, SRL vs. ERL Theory (de la Fuente, 2017), however, the final, attitudinal variable will depend on the student’s self-regulating characteristics, given that not every student has the same emotional experience with this activity:

(1) *Self-Regulation* (SR), or a *high* rating in self-regulation, has to do with a person’s positive proactivity, that is, well-adjusted, proactive management of one’s conduct (Brown, 1998). According to prior research, people have different degrees of personal self-regulation (low-medium-high), meaning the intensity and quantity of behaviors they use in regulation of their own behavior (de la Fuente, 2015b; Zapata, 2013).
(2) *A-Regulation* (AR), expressed as a *medium* rating in self-regulation, reflects a lack of proactivity or the absence of self-regulatory behaviors. Conceptually, this is equivalent to the concept of reactivity (Zimmerman and Labuhn, 2012).
(3) *Dysregulation* (DR), or a *low* rating in self-regulation, involves some amount of negative proactivity, in other words, there is active, maladjusted initiative in regulating one’s behavior. This dysregulation may have desirable side effects, such as avoiding the effort involved in proactive self-regulation, by using self-impediment strategies (Valle et al., 2007) or procrastination (Clariana, 2013; Balkis and Duru, 2017), which are ego-defensive strategies (defense of self-worth self-worth) but not good from the point of view of self-regulation.

In accordance with this typology, the SRL vs. ERL Theory predicts that: (1) students rated high in self-regulation (SR: self-regulation) would have a high degree of satisfaction with the self-assessment activity, since it offers them knowledge and tools for improvement, as well as reinforcement, given that their expected outcome and emotions are positive; (2) students with a medium rating (AR: a-regulation) would show medium satisfaction, given that the activity would involve a positive emotional sense in some cases but negative in others; (3) students with a low rating in regulation (DR: dysregulation) would have the lowest degree of satisfaction, since this activity is bothersome to them, they typically avoid it or they perform it improperly, due to expectations of a poor outcome and a negative type of emotion. This postulate is presented in Appendix 1.

### Aims and Hypotheses

Based on the previous assumptions, the general purpose of this report is: (1) research was to check whether the degree of satisfaction with the self-assessment activity of university students was related to the scores obtained and the degree of different variables, associated with level of Self-Regulation; (2) to present the online utility, *e-Coping with Academic Stress*^TM^, as a technological development in Educational Psychology; (3) analyze the possibilities of transfer of this technological innovation. More specifically, in relation to the first objective, this research was to evaluate university students’ degree of satisfaction with using *the e-Coping online utility.* The following was *hypothesized*: (1) students’ satisfaction with the self-assessment would significantly (positive or negative) correlate to the mean score obtained on the psychological variable being assessed; (2) the level of high-medium-low in Self-Regulation of students -as stated in the previous SRL vs. ERL Theory-, will significantly determine the level of each variable analyzed; (3) students’ high-medium-low level of satisfaction with self-asessment would be similar or interdependence to the high-medium-low level obtained on the each psychological variable under analysis, except for variables with reverse directionality.

## Materials and Methods

### Participants

The study sample consisted of 929 undergraduate students from a public university of the southeast (Spain). The students were enrolled in Psychology, Primary Education, or Educational Psychology; 86.5% were female (*n* = 673) and 13.5% were male (*n* = 256). The age range was 19–25 years (19 years, *n* = 201; 20 years, *n* = 303, 21 years, *n* = 131; 22 years, *n* = 82: 23 years, *n* = 49; 24 years, *n* = 38; 25 years, *n* = 20) with a mean age of 23.08 (st = 4.4) years. By academic cycles of the Degree, there were a total of 576 students of 1st cycle and 353 of 2nd cycle.

The selection of the subjects was not probabilistic, since all the students who completed the e-Coping utility were studying the subject “Educational Psychology.” The completion of the same was raised as an activity of self-evaluation and self-improvement of academic learning. Although they completed it voluntarily, by completing it they were given 2 points.

### Instruments

#### Technological Development: The Utility *e-Coping With Academic Stress*

This utility is an intervention from Educational Psychology, based on the *Competency Model for Studying, Learning and Performing under Stress* (CMSLPS; de la Fuente, 2015b). Its purpose is to help university students or professional exam candidates better manage their study, learning and related stress. This utility continues in the line of other recent intervention programs (Regehr et al., 2013), including other online interventions (Day et al., 2013). It is preferable that the instruments be used with appropriate guidance from an Educational Psychologist, in order to avoid any erroneous interpretations and inferences. In case of doubt, it is best to consult a professional. Although a good number of assessment inventories are available, *e-Coping with Academic Stress* (de la Fuente, 2015b) offers a selection of inventories that make it easier to self-assess and to enhance desired behaviors. The inventories have been validated, and the different levels were established in prior samples of university students or professional exam candidates. In any event, the levels should not be considered absolute, but merely an indication to help the student or candidate make decisions about how to improve. A low level on any particular variable, for example, would indicate the student’s need for considerable work to improve their competence in this aspect; a medium level would represent an average degree of competence, and should be improved to some extent; a high level reveals adequate competence, although certain behavioral aspects might be identified for further improvement. See Table 1.

**Table 1:** Variables of self-assessment and psychometrics characteristics of the instruments, in the e-Coping utility (de la Fuente, 2015b).

Model variables	Self-assessment	Dimensions and Reliability (our sample)	Items/Range
**Presage**			
Previous experience of stress	General Questionaireon unpleasantness(CGO)	Total (**α** = 0.73) Unpleasantness Past experience	8/1–4

**Process**			
*Meta-skills*			
Meta-cognitive	Inventory of control during study (HEME)	Total (**α** = 0.85) Before During After	44/1–5
	**Learning Approaches (2F-SPQ)**	Deep Learning (**α** = 0.81) Surface Learning (**α** = 0.77)	20/1–4
Meta-emotional	**Coping Strategies (EEC)**	Total (**α** = 0.93) Emotion-focused coping (**α** = 0.95) Problem-focused coping (**α** = 0.91)	40/1–4
Meta-behavioral	**Self-Regulation (SR)**	SR (**α** = 0.86) Goals Perseverance Decisions Learning from mistakes	20/1–4

*Skills*			
Cognitive	Learning Strategies (ECA)	Total (**α** = 0.87)	44/1–4
Emotional	Test anxiety (TAI-80)	Total (**α** = 0.91) Worry Emotionality	20/1–4
Behavioral	Note-taking (CETA)	Total (**α** = 0.84)	30/1–4
	Oral presention (CCHPO)	Total (**α** = 0.81)	30/1–4

*Attitudes*			
Achievement motivation	**TABP (JASE-H)**	Total (**α** = 0.85) Competitive-Hardworking Impatieence-Hostility	32/1–6
Resilience	**Resilience (CD-RISC)**	Total (**α** = 0.84) Tenacity Stress Change Control Spirituality	24/1–5
	**Academic Confidence (ABC)**	Total (**α** = 0.87) Grades Verbalization Study Attendance	24/1–5

**Product**			
Engagement	**(Marlach-Engagement)**	Total Engagement (**α** = 0.89) Vigor Dedication Absorption	15/1–5
Burnout	**(Utrech-Burnout)**	Total Burnout (**α** = 0.84) Exhaustion Cynicism Lack of efficacy	14/1–5

*Note: Variables used in this investigation are shown in boldface type.*

For each variable of the model, the student is able to (1) self-assess, obtain his/her own score (low-medium-high); (2) obtain improvement strategies for that variable or level of sub-competency. The tool is currently available in Spanish- and English-language versions, but it can be implemented in other languages. See Appendix 2 in Supplementary Material.

#### Instruments

##### Meta-cognitive skills for learning and study

*Learning approach.* The Revised Two-Factor Study Process Questionnaire, R-SPQ-2F (Biggs et al., 2001), in its validated Spanish version (Justicia et al., 2008), was used to measure this variable. Its 20 items pertain to four subscales (Deep Motive, Deep Strategy; Surface Motive, Surface Strategy) that measure two dimensions: Deep and Surface learning approaches, respectively. Items are scored on a 5-point Likert scale where 1 = *rarely true of me* and 5 = *always true of me*. The confirmatory factor structure of the Spanish version had a second factor structure with two factors (Chi-Square = 2645.77; df = 169, CFI = 0.95, GFI = 0.91, AGFI = 0.92, RMSEA = 0.07), which also yielded acceptable reliability coefficients (Deep, α = 0.81; Surface, α = 0.77), similar to those found by the original authors, using the AMOS Program. CFI and NFI values ranged from 0 (poor fit) to 1 (good fit; Bentler, 1990). These indices require values greater than 0.90 to represent good fit of a model. RMSEA was used because it accounts for model parsimony (i.e., goodness-of-fit values can be artificially inflated with greater numbers of parameters in the model). Our model of choice was the most parsimonious one, hence, specifying the model with a small number of parameters was preferable. RMSEA values greater than 0.08 reflect poor fit, values from 0.05 to 0.08 indicate acceptable fit, and values less than 0.05 reflect a good fit (MacCallum et al., 1996).

##### Meta-emotional skills for learning and study

*Coping Strategies Scale.* The EEC (Chorot and Sandín, 1987), in an abbreviated, validated Spanish version, EEC-Short (de la Fuente, 2014b), was used to measure this variable. While the original instrument contains 90 items, the validation revealed a first-order structure with 64 items and a second order with 10 factors and two dimensions, both of them significant and showing adequate fit values [Chi-square = 878.750; Degrees of freedom (77–34) = 43, *p* < 0.001; NFI = 0.901; RFI = 0.945; IFI = 0.903, TLI = 0.951, CFI = 0.903, RMSEA = 0.07]. Reliability measurements are Cronbach alpha of 0.93 (complete scale), 0.93 (first half) and 0.90 (second half), Spearman-Brown of 0.84 and Guttman of 0.80. Two dimensions are evaluated: (D1) Emotion-focused coping (α = 0.95); (D2) Problem-focused coping (α = 0.91). In relation to emotion-focused strategies, the factors were: (F1) Avoidant distraction (0.79); (F7) Reducing anxiety and avoidance (0.88); (F8) Preparing for the worst (0.80); (F9) Emotional venting and isolation (0.91); and (F10) Resigned acceptance (0.86). In relation to problem-focused strategies, the factors were: (F2) Seeking family help and counsel (0.92); (F5) Self-talk (0.82); (F10) Positive reappraisal and firmness (0.87); (F12) Communicating feelings and social support (0.89); and (F13) Seeking alternative reinforcements (0.80).

##### Meta-motivational variable

*Resilience.* Resilience was assessed using the *CD-RISC Scale* (Connor and Davidson, 2003) in its validated Spanish version (Mateu et al., 2010; Manzano-García and Ayala-Calvo, 2013). This inventory makes it possible to assess different aspects of being able to face difficulties and overcome them. It provides information on perceived competence, stress management, control and spirituality (Berry and York, 2010). Adequate reliability and validity values were found in Spanish samples, and a five-factor structure: F1: Persistence/tenacity, strong self-efficacy (TENACITY); F2: Emotional and cognitive control under pressure (STRESS); F3: Adaptability/ability to bounce back (CHANGE); F4: Perceived Control (CONTROL), and F5: Spirituality (SPIRITUALITY).

##### Meta-behavioral skills for learning and study

*Personal self-regulation*. The *Short Self-Regulation Questionnaire* (SSRQ) (Brown et al., 1999) was used to measure this variable. Having been previously validated in Spanish samples (Pichardo et al., 2014; Garzón-Umerenkova et al., 2017), it shows acceptable validity and reliability values, similar to the English version. The Short SRQ contains four factors (goal setting/planning, perseverance, decision making and learning from mistakes) and 17 items (all with saturations greater than 0.40), along with a consistent confirmatory factor structure (Chi-Square = 250.83, df = 112, CFI = 0.90, GFI = 0.92, AGFI = 0.90, RMSEA = 0.05). Internal consistency was acceptable for the total questionnaire (α = 0.86) and for three factors: goal setting/planning (α = 0.79), decision making (α = 0.72) and learning from mistakes (α = 0.72). The perseverance factor, however, showed low internal consistency (α = 0.63). Correlations were studied: between each item and its factor total; between the factors; and between each factor and the complete questionnaire. In all cases results were good, except in the case of decision making, which showed less correlation with the other factors (range: 0.41–0.58). The correlations between the original version and the complete version, and between the original and the short versions (complete SRQ with 32 items and short SRQ with 17 items), with a Spanish sample, were better for the short version (short-original: *r* = 0.85 and short-complete: *r* = 0.94; *p* < 0.01) than for the complete version (complete-original: *r* = 0.79; *p* < 0.01).

##### Attitudes, values and habits for learning and study

*Action-emotion style.* The *Jenkins Activity Survey for students-Form H* (JASE-H) was used. The Type-A Behavior Pattern (TABP) is measured with this scale; its student version is adapted (Bermúdez et al., 1990; Bermúdez et al., 1991, Unpublished) from the Jenkins Activity Survey T-version (Krantz et al., 1974). Its four factors are Impatience, Hostility, Competitiveness and Hardworking. There are 32 items in total, which are answered on a six-point Likert scale; the subject must choose the degree to which the item applies to him or her. A response of one means the item is not at all applicable to the respondent, and six means it is totally applicable to him or her. The JASE-H offers a global TABP score, which is the sum of scores assigned to all items, as well as specific measurements for each of the components comprising the TABP. The JASE-H possesses high internal consistency (alpha coefficient of 0.85 for the total scale; 0.81 for the Impatience-Hostility factor, 0.82 for Competitiveness and 0.70 for Hardworking) and high stability over time, both for the complete scale (0.68) and for its factors (0.61, 0.76, and 0.70, respectively). The authors report consistent Reliability and Validity measures. The statistics are Alpha = 0.832, and Guttman Split-Half = 0.803 (de la Fuente et al., 2013a).

*Academic confidence.* The *Academic Behavioral Confidence Scale*, ABC (Sander and Sanders, 2006, 2009) in a Spanish validated version (Sander et al., 2011). The ABC scale was developed from the idea that understanding students’ confidence toward their studies could be important for making sense of students’ expectations of teaching, learning and assessment. This psychometric tool assesses the confidence of under-graduate students in their own anticipated study behaviors. The ABC scale has four subscales that tap into crucially distinct aspects of students’ academic behavior: Grades, Studying, Verbalizing and Attendance (Sander and Sanders, 2009). This variable is proven to be a predictor of academic performance and learning approach (de la Fuente et al., 2013b).

##### Stress

*Emotional indicators of stress.* The *Marlach-Utrech Burnout-Engagement questionnaire* (Shaufeli et al., 2002) assesses the student’s level of engagement in the task vs. emotional exhaustion. This emotional dimension is an important correlate of subjective stress in these types of situations; there is plentiful evidence of the importance of *positive vs. negative* emotions during study, with particular importance given to the negative impact of burnout (Lorenz et al., 2010; Tavolacci et al., 2013).

### Procedure

On a voluntary basis, participants used an online platform *e-Coping Stress* (de la Fuente, 2015b; de la Fuente et al., 2015a) to complete the scales. A total of five specific teaching-learning processes were covered; they represent different university subjects that were taught over two academic years. Assessment of *presage* variables took place in September–October of 2014 and 2015, *process* variables in February–March of 2015 and 2016, and *product* variables in May–June of 2015 and 2016.

The university students used the E-Coping with Stress utility in their practicum for the subject of Educational Psychology (part of the degree programs in Psychology and Primary Education at different universities) in order to gain an understanding of the study variables through an experience with self-assessment and improvement in these variables. They completed one online questionnaire per week, at home, for 4 months. They were also assured of anonymity in data completion and data storage in a shielded database; the data were processed for the single purpose of this group investigation, never examined individually.

At the end of each inventory there is a Likert-type scale (1–5) to assess the student’s degree of satisfaction with the assessment and improvement experience afforded by the e-utility for that variable. This scale is completed just after finishing each inventory, before learning one’s score. The score on this final scale revealed users’ subjective satisfaction with each assessment, as well as their specific experience with each inventory, making it possible to detect which one is most useful, as well as the optimal moment for application during university studies.

The students participated voluntarily an informed consent from the participants was obtained and was previously approved by the University Ethics Committee each university (Bioethics Committee of the University of Almería, and Commission of Research Ethics of the University of Navarra), in the context of R&D Project (2012-2015). The consent obtained was both informed and written. The data was protected in an archived and registered file, as indicated by the Spanish Data Protection Law.

### Data Analysis

Using an ex-post-facto design, preliminary ANOVAS were carried out to rule out an effect of age, gender and academic cycle on satisfaction with the self-assessment. First, a bivariate correlation of Pearson was analyzed. In a complementary way, the descriptive indexes of each variable were calculated (means, standard deviation, asymmetry and kurtosis). Second, ANOVAS and MANOVAS were carried for determination of effect of the level of Self-regulation (high-medium-low) on level of each variable. Third, low-medium-high groups were established for each of the variables through a K-means cluster analysis. Several ANOVAs were also carried out, to establish independence between low-medium-high levels of each variable and from the level of satisfaction with the *self-assessment activity*. Statistical suitability of these groupings was also established by ANOVAs, as well as the effects of the dependent variables, using SPSS v. 23.0.

## Results

### Preliminary Results: Effect of Gender and Cycle on Satisfaction With Self-Assessment Activity

The previous ANOVAS carried out showed non-significant main effects of the *gender* variables [*F*(1,831) = 2.635, *p* < 0.123, η^2^ = 0.003, power = 0.370] and *academic cycle* [*F*(1,831) = 1.769, *p* < 0.172, η^2^ = 0.002, power = 0.264], in the *satisfaction* with the self-assessment carried out.

### Descriptive Values and Association Relations Between Satisfaction With the Self-assessment, and Variable Assessed (Hypothesis 1)

The results showed significant bivariate correlations of the positive dimensions of the variables analyzed and satisfaction with the self-assessment. A significant, positive bivariate correlation appeared between Satisfaction with Self-Asssessment (SAT) and the following: Self-Regulation (SR) (*r* = 0.275), Deep Approach (DA) (*r* = 0.324), Problem Coping (CP) (*r* = 0.178), Resilience (*r* = 0.312), the Competence-Hardworking (CHW) component (*r* = 0.538), Academic Confidence (AC) (*r* = 0.333) and Engagement (*r* = 0.275). Also, a significant negative relationship appeared between the negative dimensions of the variables in relation to Surface Approach (SA) (*r* = -0.114) and Burnout (*r* = -0.375). See Table 2.

**Table 2:** Correlation and mean values of the variables analyzed (*n* = 929).

*Variable*	*SAT*	*Mean (SD)*	*Range*	*Asymmetry*	*Kurtosis*
*Meta-behavioral*					
Self-Regulation (SR)	0.275^∗∗^	3.86 (0.811)	1.98–5.00	0.566 (0.105)	0.799 (0.209)
*Meta-cognitive*					
Deep approach (DA)	0.324^∗∗^	3.62 (0.831)	1.30–4.40	0.089 (0.106)	–0.444 (0,211)
Surface approach (SA)	–0.114^∗∗^	3.62 (0.831)	1.00–4.40	0.696 (0.106)	0.650 (0.211)
*Meta-emotional*					
Total coping strategies	0.167^∗∗^	3.71 (1.01)	1.43–4.00	0.493 (0.083)	–1.02 (0.165)
Emotional coping (EC)	0.048	3.32 (1.38)	1.23–4.00	0.508 (0.081)	–1,04 (0.162)
Problem copin (PC)	0.178^∗∗^	3.37 (1.44)	1.46–4.00	0.236 (0.080)	–1.01 (0.160)
*Meta-motivational*					
Resilience (RES)	0.312^∗∗^	3.89 (0.870)	2.20–4.80	–0.051 (0.128)	0.160 (0.225)
*Attitudinal*					
Action-Emotion Style (AES)	0.527^∗∗^	3.40 (1.25)	1.16–5.29	0.273 (0.086)	0.120 (0.171)
Competitiv-Hardworking (CH)	0.538^∗∗^	3.52 (1.10)	1.25–5.60	0.196 (0.085)	–0.204 (0.169)
Impatience-Hostility (IH)	–0.435^∗∗^	3.61 (1.32)	1.10–5.43	0.343 (0.085)	0.001 (0.170)
Academic Confidence (AC)	0.333^∗∗^	3.87 (0.890)	2.00–5.00	–0.246 (0.117)	0.363 (0.234)
*Estres*					
Engagement (ENG)	0.275^∗∗^	3.98 (0.086)	1.60–4.72	–0.255 (0.304)	0.025 (0.599)
Burnout (BURN)	–0.375^∗∗^	3.98 (0.086)	1.13–4.13	0.696 (0.311)	–0.267 (0.613)

*SAT, Satisfaction with Self-Asessment; DA, Deep Approaches; SA, Surface Approaches; SR, Self-Regulation; CTOT, Coping Total; EMOTC, Emotion Coping; PROBC, Problem Coping; RESIL, resilience; AES, Action-Emotion Style; CSL, Competivity-Hard Word; IH, Impatience-Hostility; AC, Academic Confidence; BURN, Burnout; ENGA, Engagement.*

### Effect of the High-Medium-Low Level of Self-Regulation on HML Level of Each Variable (Hypothesis 2)

The results showed that in most of the analysis, the level of self-regulation of the students (high-medium-low) determines, in a statistically significant way, the level of the variable analyzed. Initially it was found that the levels established in Self-Regulation (High-Medium-Low, HML) were significantly different.

First, for the *meta-cognitives* variables, a significant main effect of the HML level of SR appeared in the dimensions of the learning approaches for both dimensions, although in the opposite direction. The HML levels in SR determine the same levels of Deep Approaches (DA) and the inverse in Surface Approaches (SA). Fort the *meta-emocional* variables, in the case *of coping strategies, although* a significant main effect did not appear in the total number of strategies used, a significant partial effect did appear for the problem-centered strategies, for the former students and the media in self-regulation. Also, the HML level of SR determined the levels of *meta-motivational* variable Resilience.

In the case of *attitudinal* variables, the HML level of SR significantly determined the HML level of the emotion-action style (directly with respect to the CH dimension, and infiractly with respect to the IH dimension) and of *academic confidence.*

Finally, for the variables of *stress*, the HML level of SR positively determined the level of engagement and negatively the level of the burnout variable. The direct mean values and the specific effects are shown in Table 3.

**Table 3:** Effect of the level of *Self-Regulation* (high-medium-low) (IV) on level of each variable (DVs) (*n* = 929).

*Variable*	*High SR (H)* (*n* = 276) *Regulation*	*Medium SR (M)* (*n* = 425) *A-Regulation*	*Low SR (L)* (*n* = 228) *Dys-Regulation*	*Effect (Pillai’s test)*	*Post-hoc*
*Meta-behavior*					
SR	3.54 (1.19)^∗^	3.39 (0.20)	3.27 (0.22)	*F*(2,927) = 47.80^∗∗^, η^2^= 0.165, power = 1.0	H > M > L^∗∗^
*Meta-cognitive*					
DA	3.10 (0.696)^∗^	2.85 (0.609)	2.60 (0.635)	*F*(2,927) = 5.939^∗∗^, η^2^= 0.028, power = 0.88	H > M > L^∗∗^
SA	2.02 (0.536)	2.23 (0.668)	2.30 (0.551)^∗^	*F*(2,927) = 4.831^∗∗^, η^2^= 0.023, power = 0.79	L > M, H^∗∗^
*Meta-emotional*					
CTOT	2.74 (0.35)^∗^	2.64 (0.37)	2.67 (0.36)	*F*(2,927) = 1.348 ns, η^2^= 0.006, power = 0.291	
EMOTC	2.51 (0.30)	2.44 (0.29)	2.72 (0.31)^∗^	*F*(2,927) = l.133 ns, η^2^= 0.005, power = 0.250	L > M, H^∗^
PROBC	2.98 (0.32)^∗^	2.84 (0.29)	2.42 (0.30)	*F*(2,927) = 2.830^∗∗^, η^2^= 0.030, power = l.00	H, M > L^∗∗^
*Meta-motivational*					
RESIL	3.78 (0.46)^∗^	3.53 (0.43)	3.42 (0.41)	*F*(2,927) = 13.160^∗∗^, η^2^= 0.081, power = 0.997	H > M, L^∗∗^
*Attitudinal*					
AES	3.64 (0.55)^∗^	3.35 (0.38)	3.21 (0.57)	*F*(2,927) = 3.469^∗^, η^2^= 0,014, power = 0.648	L < M, L^∗^
CHW	3.89 (0.68)^∗^	3.63 (0.70)	3.44 (0.73)	*F*(2,927) = 5.272^∗∗^, η^2^= 0.021, power = 0.83	H, M > L^∗∗^
IH	2.81 (0.68)	3.02 (0.76)	3.18 (0.77)^∗^	*F*(2,927) = 2.278^∗^, η^2^= ,011, power = .548	H,M < L^∗∗^
AC	4.00 (0.41)^∗^	3.77 (0.44)	3.57 (0.46)	*F*(2,927) = 20.303^∗∗^, η^2^= 0.102, power = 1.0	H > M > L^∗∗^
*Stress*					
ENGA	3.42 (0.57)^∗^	3.31 (0.63)	2.86 (0.71)	*F*(2,927) = 2.796^∗^, η^2^= 0.113, power = 0.553	H, M > L^∗^
BURN	2.05 (0.49)	2.35 (0.83)	2.56 (0.67)^∗^	*F*(2,927) = 2.541^∗^, η^2^= 0.030, power = 0.157	H, M < L^∗^

*SR, Self-Regulation; DA, Deep Approaches; SA, Surface Approaches; CTOT, Coping Total; EMOTC, Emotion Coping; PROBC, Problem Coping; RESIL, Resilience; AES, Action-Emotion Style; CHW, Competivity-Hard Word; IH, Impatience-Hostility; AC, Academic Confidence; BURN, Burnout; ENGA, Engagement; ^∗^significant major trend; ^∗∗^*p <* 0.001.*

### Effect of the High–Medium–Low Level of Each Variable on HML Satisfaction With the Self-Assessment Experience (Hypothesis 3)

The high–medium–low levels of the variables analyzed showed different significant effects on satisfaction with the self-assessment (SAT). Students’ levels of self-regulation, as a meta-behavioral variable, determined their level of SAT, and also their level of learning approaches, whether positively (DA) or negatively (SA). Likewise, the level of the meta-emotional variable coping strategies determined the level of SAT, especially in the case of problem-focused coping, but not in emotion-focused coping. Accordingly, the same occurred for levels of resilience (RES), action-emotion style (AES) and academic confidence (AC). Finally, the level of the variable burnout (BUR) determined the level of SAT; the inverse was true for level of engagement (ENG). See Table 4.

**Table 4:** Differences according the level of the variable assessed (IV) on the level of Satisfaction with the Self-Assessment, SAT (DV) (*n* = 929).

*Variable*	*High (H)* (*n* = 276)	*Medium(M)* (*n* = 425)	*Low (L)* (*n* = 228)	*Effect (Pillai test)*	*Post-hoc*
*Meta-behavior*					
SR	3.93 (1.12)^∗^	3.69 (1.24)	3.31 (1.28)	*F*(2,927) = 20.56^∗∗^, η^2^= 0.035, power = l.0	H > M > L^∗∗^
*Meta-cognitive*					
DA	3.97 (0.786)^∗^	3.61 (0.759)	3.27 (0.868)	*F*(2,927) = 49.23^∗∗^, η^2^= 0.088, power = l.0	H > M > L^∗∗^
SA	3.49 (0.862)	3.53 (0.800)	3.73 (0.810)^∗^	*F*(2,927) = 8.72^∗∗^, η^2^= 0.017, power = 0.89	L > M, H^∗∗^
*Meta-emotional*					
CTOT	2.99 (1.56)^∗^	3.45 (1.37)	3.52 (1.49)	*F*(2,927) = 7.638^∗∗^, η^2^= 0.020, power = 0.947	H < M,L ^∗∗^
EMOTC	3.25 (1.43)	3.43 (1.25)	3.41 (1.43)^∗^	*F*(2,927) = l.68, ns, η^2^= 0.004, power = 0.335 n.s.	H,M > L^∗∗^
PROBC	3.55 (1.44)^∗^	3.42 (1.41)	3.07 (1.14)	*F*(2,927) = 7.04^∗∗^, η^2^= 0.015, power = l.00	
*Meta-motivational*					
RESIL	2.78(2.10)^∗^	2.41 (1.91)	2.17 (1.79)	*F*(2,927) = 7.09^∗∗^ η^2^ = 013, power = 0.931	H,M > L^∗∗^
*Attitudinal*					
AES	3.74 (1,18)^∗^	3.58 (1.27)	3.39 (1.46)	*F*(2,927) = 4.323^∗^, η^2^= 0.023, power = 0.998	H > M,L^∗∗^
CHW	3.93 (1.30)^∗^	3.63 (1.25)	3.26 (1.38)	*F*(2,927) = 5.356^∗^, η^2^= 0.038, power = l.0	H > M > L^∗∗^
IH	3.58 (1.40)	3.54 (1.29)	3.86 (1.17)^∗^	*F*(2,927) = 4.256^∗^, η^2^= 0.008, power = 0.745	L > M, H^∗∗^
AC	3.98 (1.27)^∗^	3.27 (1.33)	3.07 (1.35)	*F*(2,927) = 39.50^∗∗^, η^2^= 0.067, power = 1.0	H > M > L^∗∗^
*Stress*					
ENGA	4.37 (0.71)^∗^	3.95 (0.79)	3.52 (0.95)	*F*(2,927) = 64,68^∗∗^, η^2^= 0.125, *p* = l.0,	H > M > L^∗∗^
BURN	3.56 (0.98)	3.88 (0.79)	4.29 (0.73)^∗^	*F*(2,927) = 56,201^∗∗^, η^22^= 0.108, *p* = l.0,	H < M < L^∗∗^

*SR, Self-Regulation; DA, Deep Approaches; SA, Surface Approaches; CTOT, Coping Total; EMOTC, Emotion Coping; PROBC, Problem Coping; RESIL, resilience; AES, Action-Emotion Style; CHW, Competivity-Hard Word; IH, Impatience-Hostility; AC, Academic Confidence; BURN, Burnout; ENGA, Engagement; ^∗^Significant major trend; ^∗∗^*p* < 0.001.*

## Discussion and Conclusion

### Research

The results confirming the *first hypothesis* of our investigation contributed empirical support for the idea that satisfaction of self-assessment activities (SAT) is associated with the variables assessed. If we consider that self-assessment is a behavior typical of self-regulation in its different phases (before, during, and especially after the behavior), these results are consistent with prior results that have consistently shown self-regulation to be linearly and positively predictive of flourishing and health, while a linear, negative prediction is found for reasons to procrastinate (Garzón-Umerenkova et al., 2018), and the use of motivational regulation strategies had significant positive indirect effects on students’ academic performance and affective/cognitive well-being (Grunschel et al., 2016). There is also a significant positive predictive relationship between self-regulation and resilience, with the self-regulation factor learning from mistakes being the most predictive of resilience (Artuch-Garde et al., 2017). Likewise, a positive interdependence relationship is found between level of self-regulation, deep learning approaches, problem-focused coping strategies, resilience, and academic confidence, with a negative interdependence relationship for test anxiety (de la Fuente et al., 2017).

One relevant aspect of the results is the negative association found between the activity of self-assessment and surface learning approaches and between self-assessment and burnout. If students with these characteristics manifest dissatisfaction with the activity of self-assessment, it is probably because the activity itself can induce negative emotionality and stress when results are less than adequate or are indicative of the student’s low skills for learning. This may be at the root of this association; however, this aspect should be further investigated. This matter should also be taken into account in university student advising and guidance processes. Help with self-assessment, or an online self-help program, is not sufficient; mentoring programs are needed that teach these students to continue self-improvement without becoming discouraged, and offer them external support.

The *second hypothesis* afirmed the level of high-medium-low in Self-Regulation of students will significantly determine the level of each variable analyzed. This results they show empirical support, since it was proved that the HML levels of SR determine the HML levels of the analyzed variables, as stated in the previous SRL vs. ERL Theory (de la Fuente, 2017; see Appendix 1), and recent empirival evidence (de la Fuente et al., 2017). Once again, the importance of personal self-regulation as a meta-behavioral correlate of the subjects that influences the rest of the variables, meta-cognitive, meta-emotional, meta-motivational, and stress level is confirmed.

The *third hypothesis* asserted that high levels in the independent variables that form part of Self-Regulated behavior (SR) would be accompanied by high satisfaction with the performance of SAT. By contrast, low levels in the independent variables, representing low regulation or dysregulation (DR), would be accompanied by the lowest level of satisfaction with the self-assessment experience. In the case of self-regulating students (SR), as the data confirm, this system of self-assessment and self-help is optimal, considering that these students are characterized by personal work and they are able to take best advantage of an autonomous self-help system. In the case of students who are medium and -especially- low in self-regulation (dysregulatory), the evidence moves us closer to an explanatory mechanism for why such students avoid doing self-assessment, or simply put it off with procrastination (Garzón-Umerenkova et al., 2018) or distractive mechanisms; self-assessment generates further stress for them, in addition to what they already experience.

However, these results have the *limitation* that they are not generalizable to the entire population because they have not used probabilistic sampling and are limited to the students who participated in the use of the e-Utility. Future research should show if this effect is maintained with all types of university students, from different countries and cultures.

### Implications for Intervention

Consequently, the “Matthew effect” (Merton, 1968) would be applicable in their case, where university students with low regulation or dysregulation gain no satisfaction from doing improvement-oriented self-assessments; leading them to even further dysregulation, considering that self-assessment is a mechanism that compiles critical information for the exercise of self-regulation. From our point of view, this represents an important *limitation* of this e-utility, given that when used alone, as is the case, without external regulation (in time and extrinsic motivation), there is a high likelihood that the self-assessment and improvement tasks will be dropped at some point. It is therefore more advisable to use them in a context of external regulation that engages the students and ensures their completion. Advisory support for their use is also of benefit, in order to ensure their profitability.

On the other hand, there is a growing academic interest in the development of preventive or remedial interventions, virtual or face-to-face, that support the academic quality of the institutions and the achievement of goals by the students. For example, for the management of academic procrastination (Rozental et al., 2014; De Paola and Scoppa, 2015; Glick and Orsillo, 2015), time management (Nadinloyi et al., 2013), stress reduction (Regehr et al., 2013), promotion of health behaviors (Webb et al., 2010) or improvement of academic performance (Lin and Tsai, 2016). The focus on cognitive-behavioral change through the different options offered by the internet is a source of research into R & D programs with social impact (Webb et al., 2010) that, as in the research presented, point to a path for the development of this type of evaluation or feedback tools with verifiable results.

For some years, the lack of application of the principles and results of educational research in the context of educational practice has been worrisome (Vanderlinde and van Braak, 2010; Levin, 2013). The development presented “e-utility” allows to facilitate the application of theoretical principles of education to improvements for practical purposes, in different academic contexts or even from other contexts in which the same principles can be transferred, such as working environments (Rabenu and Yaniv, 2017) or personnel selection processes (Sautelle et al., 2015).

### Theoretical Implications

When learning and study involve pressure and potentially stressful situations, emotional experiences are just as important as the cognitive processes used. Traditionally, in its effort to help students, the psychological assessment of study and learning has focused on cognitive skills and metacognitive strategies. This approach is reasonable when learning contexts are not stressful. In contexts of university teaching and learning, however, conditions often trigger stress responses. Such conditions include highly demanding tasks, high performance requirements, sustained effort and uncertainty about succeeding in one’s objectives. In such cases, not only cognitive behaviors must be examined, but also emotional behaviors. In the case of professional examination candidates, work must be approached from a *competency model for competing in professional examinations* (de la Fuente, 2015a), integrating the conceptual, procedural and attitudinal levels of the subcompetencies. The utility *e-Coping with Academic Stress* (de la Fuente, 2015b) - based on the SLPS Competency Model- offers students the opportunity to self-assess their achievement emotions, and subsequently work toward self-improvement, at different points of the teaching-learning process. Teachers may also benefit by understanding the levels of these variables that are represented in their students and their class groups, and by making suitable adjustments in the teaching-learning process.

### Implications for the Transfer of e-Utility

From the conception of the value chain RD & I, it is important to carry out activities of transfer of the empirically validated theoretical models and technological developments arising in the field of Educational Psychology. Different innovation transfer activities were carried out and are presented here. They are based on the *SLPS Competency Model^®^*, for learning how to learn, and the *E-Coping with Academic Stress^®^* utility:

#### Transfer Seminars

This innovation transfer activity consists of presenting the model, the technology developed, and its possible applications in different healthcare and educational organizations. Several actions of this type have been carried out, in hospitals as well as in Spanish and other European universities.

##### Catalog of Public Purchases in Innovation

The *e-Coping with Academic Stress^®^* utility was included in the Catalog of Public Purchases in Innovation, prepared by the AMETIC Technology Platform and the Ministry of Health (Spain). The catalog includes technology developments typical of E-Healthcare and seeks to promote the use of new technology developments from companies in the sphere of public and private healthcare: http://ides.es/blog/cat%C3%A1logo-de-oferta-innovadora-en-tics-de-la-salud-vida-activa-e-independiente.

#### R&D Technology Transfer Contracts

R&D transfer contracts of technological innovation were carried out with two European universities. In each case, the contract included general consulting and improvement of the R&D&I value chain, and in particular, use of the models and technological tools mentioned above.

#### Transfer to UAL Spin-Off Company, Education and Psychology I + D + i

The university’s office of research results transfer has transferred these products to the cited company for their exploitation.

#### Technological Demonstrations

More recently, technological demonstrations have been held at conferences on Psychology and Technological Innovation and Entrepreneurship (de la Fuente et al., 2018b): http://cipi2018.copao.com/es/.

## Author Contributions

JdlF: utility development, research design, data analysis, and text writing. JM-V: data analysis and text revision. FP-S: completion of the students. MG-T: completion of the students and general revision of the text. RA: sample collection and bibliographic review. AG-U: general revision of the English version in the text.

## Conflict of Interest Statement

The authors declare that the research was conducted in the absence of any commercial or financial relationships that could be construed as a potential conflict of interest.

## Appendix 1

**Table A1:** Conceptual continuum and typologies of each Self-Regulatory Behavior (reproduced with permission: de la Fuente, 2017).

Characteristics of the person	Self-Regulation (SR) *High Self-Regulation* POSITIVE PRO-ACTIVITY (+1)	A-Regulation (AR) *Medium Self-Regulation* RE-ACTIVITY (0)	Dysregulation (DR) *Low Self-regulation* NEGATIVE PRO-ACTIVITY (-1)
	*Before* Self-analysis of tasks Self-defines goals Self-motivation	*Before* No analysis of tasks No goals No motivation	*Before* Erroneous self-analysis Erroneous goals Self-demotivation
	*During* **Self-observation^∗^ Self-analysis Self-correction**	*During* **No self-observation^∗^ No supervision No self-correction**	*During* **Self-distraction^∗^ Cognitive self-avoidance Self-impediment Procrastination**
	*After* **Self-reflection^∗^ Self-attributions Positive self-affects**	*After* **No reflection^∗^ No attributions No affects**	*After* **Erroneous self-assessment^∗^ Erroneous self-attributions Negative self-affect**
**Type of Activity**	**Self-Regulatory (SR) High-Moderate-Low PRO-ACTIVITY (+)**	**A-Regulatory (AR) No regulation RE-ACTIVITY (=)**	**Dysregulatory (DR) Low-Moderate- High PRO-ACTIVITY (-)**
Academic	Self-regulated learning	No norms/limits	Self-induction impediment
Road safety	Self-regulation in driving	No norms/limits	Self-induction of risks
Health	SR in Health	No norms/limits	Self-induction of excesses
TV	SR in TV	No norms/limits	Self-induction of excesses
Family	SR in family	No norms/limits	Self-induction of risks
Technology of Information and Communication (TIC)	SR in TIC	No norms/limits	Self-induction of excesses
Sexual	SR in risky sexual behavior	No regulation	Self-induction of risks
Violence	SR in harmonious relations	No norms/limits	Self-induction of excesses
Spouse/partner	SR in interaction	No regulation	Self-induction of excesses

*^∗^Place of self-assessment and positive vs. negative self-affect.*
