# Supplementary material for: Satisfaction With the Self-Assessment of University Students Through e-Coping With Academic Stress UtilityTM
Source: Front Psychol. 2018 Nov 8;9:1932. doi: 10.3389/fpsyg.2018.01932 (PMC6236068; doi:10.3389/fpsyg.2018.01932)
Supplement: Supplementary file 1 [file Data_Sheet_1.docx]

**APPENDIX 2. COMPLEMENTARY MATERIAL**

*Figure 2.* Completion of an inventory. *Note:* student name is simulated

Figure 3. Results from an inventory. *Note:* student name is simulated.

Figure 4. Improvement strategies for one inventory. *Note:* student name is simulated.
